# Comprehensive Pan‐Cancer Analysis of TRNT1 as a Potential Biomarker for Breast Cancer

**DOI:** 10.1111/jcmm.70853

**Published:** 2025-09-26

**Authors:** Xinwei Li, Yue Meng, Bing Gu

**Affiliations:** ^1^ Guangdong Cardiovascular Institute Guangdong Provincial People's Hospital (Guangdong Academy of Medical Sciences), Southern Medical University Guangzhou China; ^2^ Laboratory Medicine Guangdong Provincial People's Hospital (Guangdong Academy of Medical Sciences), Southern Medical University Guangzhou China; ^3^ Guangdong Provincial Clinical Research Center for Laboratory Medicine Guangzhou China

**Keywords:** biomarker, breast cancer, prognosis, TCGA, TRNT1

## Abstract

TRNT1, an RNA nucleotide transferase, plays a critical role in cellular processes and may be involved in cancer. However, its role in cancer has not been fully explored. This study aims to explore the potential significance of TRNT1 in cancer, particularly in breast cancer (BC) progression and prognosis. A pan‐cancer analysis of TRNT1 was conducted using various online bioinformatics tools, including GEPIA2, cBioPortal, TIMER2, Metascape, and UALCAN, combined with experimental validation. The analysis encompassed gene expression, prognosis, genomic alterations, immune infiltration, and functional enrichment. Additionally, in vitro experiments were performed to further investigate TRNT1's role in BC. TRNT1 is highly expressed in most cancers, with significant correlation to prognosis, especially in BC. Promoter methylation and genomic alterations may contribute to its abnormal expression. Furthermore, functional enrichment analysis revealed that TRNT1‐associated genes are primarily involved in protein processing and RNA metabolism. Our study also represents the first evidence that TRNT1 is overexpressed in BC, participates in tumour proliferation, and may regulate apoptosis through the P53 pathway. This initial pan‐cancer study provides a relatively comprehensive understanding of the oncogenic role of TRNT1 across various cancers and highlights its potential as a biomarker for BC.

**Abbreviations**
BC: breast cancer
BLCA: bladder urothelial carcinoma
cBioPortal: cancer genomics portal
CHOL: cholangiocarcinoma
COAD: colon adenocarcinoma
CPTAC: clinical proteomic tumour analysis consortium
ESCA: oesophageal carcinoma
ESCC: oesophageal squamous cell carcinoma
GEPIA2: gene expression profiling interactive analysis (version 2)
GO: gene ontology
HNSC: head and neck squamous cell carcinoma
HPA: human protein atlas
IHC: immunohistochemical
KEGG: Kyoto encyclopedia of genes and genomes
KICH: kidney chromophobe
KIRC: kidney renal clear cell carcinoma
KIRP: kidney renal papillary cell carcinoma
LIHC: liver hepatocellular carcinoma
LUAD: lung adenocarcinoma
OC: ovarian cancer
OS: overall survival
PAAD: pancreatic adenocarcinoma
PCPG: pheochromocytoma and paraganglioma
PRAD: prostate adenocarcinoma
RCC: renal cell carcinoma
READ: rectum adenocarcinoma
SARC: sarcoma
STAD: stomach adenocarcinoma
STRING: search tool for the retrieval of interacting genes/proteins
TCGA: the Cancer Genome Atlas
THCA: thyroid carcinoma
TIME: tumour immune microenvironment
TIMER2.0: tumour immune estimation resource (version 2.0)
TISIDB: tumour immune single‐cell expression database
TLS: tertiary lymphoid structures
TMAs: tissue microarrays
UALCAN: University of Alabama at Birmingham Cancer Data Analysis Portal
UCEC: uterine corpus endometrial carcinoma

## 1 Introduction

TRNT1 (tRNA nucleotidyltransferase 1) is an RNA transferase encoded by the TRNT1 gene, located on human chromosome 3 at position 26.2, with a gene length of approximately 20 kb [1]. The primary function of TRNT1 is to catalyse the addition of a Cytosine‐Cytosine‐Adenine (CCA) trinucleotide at the 3′ end of cytoplasmic and mitochondrial tRNAs during post‐transcriptional modification. This modification is essential for proper aminoacylation of tRNA, accurate ribosome positioning, and subsequent protein synthesis [2].

Despite the critical biological functions of TRNT1, its role in human diseases has only recently attracted considerable attention [3]. Mutations in the TRNT1 gene have been implicated in several disorders, including congenital sideroblastic anaemia with immunodeficiency, retinal pigment degeneration with microcytic anaemia (SIFD), and adult‐onset progressive B‐cell immunodeficiency [4, 5]. Moreover, TRNT1 has been implicated in oncogenesis through gene expression studies of colorectal cancer. A comparison of gene expression profiles between African American and Caucasian American colorectal cancer patients revealed significantly lower expression of TRNT1 in the African American cohort, which is associated with higher incidence and mortality rates of colorectal cancer in this population [6]. These findings suggest that TRNT1 may play a significant role in the onset and progression of cancer. However, the expression patterns, prognostic significance, and molecular mechanisms of TRNT1 in most cancer types remain underexplored.

Breast cancer (BC) is the most common cancer worldwide and a leading cause of cancer‐related mortality among women [7]. The pathogenesis of breast cancer is complex and multifactorial [8]. Given the complexity of tumourigenesis, analysing the expression of genes of interest and evaluating their correlation with prognosis and underlying molecular mechanisms hold considerable potential. Therefore, it is critical to further investigate the role of TRNT1 in malignant tumours, including breast cancer.

In this study, we performed a comprehensive analysis of TRNT1, encompassing gene expression, protein expression, survival outcomes, mutations, DNA methylation, immune‐related analysis, and gene set enrichment analysis. We assessed its association with prognosis and explored potential molecular mechanisms. Additionally, we used breast cancer tissue microarrays and various in vitro cell‐based experimental techniques to investigate the biological function of TRNT1 in breast cancer and the signalling pathways it may involve.

## 2 Materials and Methods

### 2.1 Expression Analysis

The Human Protein Atlas (HPA) database (https://www.proteinatlas.org/) was utilised to investigate the mRNA expression levels of TRNT1 across normal human tissues [9]. To assess the differential expression of TRNT1 in various tumour versus normal tissues, the Tumour Immune Estimation Resource (TIMER2.0) (http://timer.cistrome.org/) was employed [10]. Additionally, the University of Alabama at Birmingham Cancer (UALCAN) tool (https://ualcan.path.uab.edu/) was used to analyse the differential expression of total protein levels of TRNT1 in tumour and normal tissues, drawing on datasets from the Clinical Proteomic Tumour Analysis Consortium (CPTAC) [11].

### 2.2 Survival Prognosis Analysis of TRNT1

The Kaplan–Meier Plotter (http://kmplot.com/analysis/) was employed to explore the relationship between TRNT1 expression and overall survival (OS) in patients with various tumours, including BC, Oesophageal Squamous Cell Carcinoma (ESCC), Kidney Renal Clear Cell Carcinoma (KIRC), and Liver Hepatocellular Carcinoma (LIHC) [12]. For patient expression stratification, the ‘Auto select best cutoff’ option was utilised.

### 2.3 Genetic Alteration Analysis

We accessed the cBioPortal website (https://www.cbioportal.org/) [13], and selected ‘TCGA Pan Cancer Atlas Studies’ using the ‘Quick select’ function to investigate the genetic alteration characteristics of TRNT1. The mutation frequency across all tumour types in the Cancer Genome Atlas (TCGA) database was visualised using the ‘Cancer Types Summary’ module. Information regarding the mutation sites of TRNT1 was obtained through the ‘Mutations’ module. Additionally, the ‘Comparison’ module was utilised to acquire data on overall, disease‐free, progression‐free, and disease‐specific survival differences in cancer cases with or without alterations in the TRNT1 gene. Kaplan–Meier plots were generated for visualisation.

### 2.4 DNA Promoter Methylation Analysis

Promoter methylation levels between various TCGA cancer types and their corresponding normal tissues were evaluated using the methylation module available on the UALCAN platform. Box plots were generated to visualise the methylation data.

### 2.5 Immune Infiltration Analysis

The correlation between TRNT1 expression and the tumour immune microenvironment (TIME) was analysed using the Tumour Immune Single‐cell Expression Database (TISIDB) (http://cis.hku.hk/TISIDB/) [14]. This analysis encompassed tumour‐infiltrating lymphocytes, immunoinhibitors, and immunostimulators. Data visualisation was accomplished through the generation of heatmaps and scatter plots.

### 2.6 TRNT1‐Related Gene Enrichment Analysis

We first performed a search for the protein name ‘TRNT1’ and the organism “ *Homo sapiens* ” on the STRING database (https://string‐db.org/) [15]. The following key parameters were configured: the minimum required interaction score was set to ‘Low confidence (0.150),’ the meaning of network edges was set to ‘evidence,’ the maximum number of interactors to show was limited to ‘no more than 50 interactors’, and the active interaction sources were restricted to ‘experiments.’ Consequently, we identified the experimentally determined proteins related to TRNT1. Gene Expression Profiling Interactive Analysis (GEPIA2) was employed to identify the top 100 genes positively associated with TRNT1 expression based on datasets from all TCGA tumours and normal tissues [16]. Additionally, the ‘Gene_Corr’ module of TIMER 2.0 was then employed to generate a correlation heatmap for these genes. For further functional analysis, the Metascape tool (https://metascape.org/gp/index.html) was utilised to perform Gene Ontology (GO) and Kyoto Encyclopedia of Genes and Genomes (KEGG) analyses [17].

### 2.7 Immunohistochemical (IHC) Staining

Tissue microarrays (TMAs) were used for IHC staining. The TMAs, obtained from Shanghai Outdo Biotech Co. Ltd., included 139 breast cancer tissue samples and 26 adjacent non‐cancerous tissue samples. For the staining procedure, tissue sections were incubated overnight at 4°C with a rabbit polyclonal antibody against TRNT1 (Thermo Fisher Scientific, 1:800). Following this, sections were incubated with a secondary antibody. The staining reaction was developed using 3,3′‐diaminobenzidine (DAB) as the chromogen. The stained tissue sections were then examined and photographed under a microscope. Quantitative analysis of the IHC‐stained sections was performed using ImageJ software with the IHC Profiler plugin. The histoscore (H‐score) was used to assess the expression of TRNT1. The H‐score was calculated by assessing both the intensity of staining and the percentage of positive tumour cells. Staining intensity was graded on a scale of 0 to 3: 0 (negative), 1 (Low Positive), 2 (Positive), and 3 (High Positive). H‐score = ∑(PI × I) = (Low Positive Cell Percentage×1) + (Positive Cell Percentage × 2) + (High Positive Cell Percentage × 3) [18].

### 2.8 Cell Culture and Transfection

MCF‐7 and T‐47D cell lines were sourced from Guangzhou Cellcook Biotech, while MCF‐10A, BT‐549, and SK‐BR‐3 were obtained from Procell Life Science. MCF‐10A cells were cultured in a specialised medium (Procell), MCF‐7 in DMEM with 10% FBS and 1% antibiotics (Gibco), BT‐549 and T‐47D in RPMI‐1640 with 10% FBS and 1% antibiotics (Gibco), and SK‐BR‐3 in McCoy's 5A with 10% FBS and 1% antibiotics (Procell). All cell lines underwent STR profiling for authentication and were regularly screened for mycoplasma.

siRNAs, including si‐NC and si‐TRNT1, were purchased from RiboBio. Transfections were performed using Lipofectamine 3000 (Invitrogen) as per the supplier's guidelines. TRNT1 siRNA sequences were: #1 (5′‐GGATTCGGATGATAAACAA‐3′), #2 (5′‐GAAAAACCTTGGCTTATTT‐3′), #3 (5′‐GGAACCTGATGCAACTACT‐3′). MCF‐7 cells were plated and transfected at 30%–50% confluency using 50 pmol siRNA per 10^5^ cells, harvesting cells after 72 h for Western blot. Lentiviral packaging was conducted by VectorBuilder. Transduced MCF‐7 and SK‐BR‐3 cells were cultured with 1.0 μg/mL puromycin for 72 h and then collected for downstream experiments.

### 2.9 Western Blot

Proteins were extracted from cells using RIPA buffer (Solarbio) with the addition of 1% protease inhibitor (Biotech) and 1% phosphatase inhibitor mixtures (Biotech). Protein concentrations of the samples were determined using the BCA assay. Sample proteins were separated via sodium dodecyl sulfate‐polyacrylamide gel electrophoresis (SDS‐PAGE) and subsequently transferred onto nitrocellulose membranes (Millipore). Membranes were blocked with 5% nonfat milk at room temperature for 1 h. Following blocking, membranes were incubated overnight at 4°C with specific primary antibodies. The membranes were then incubated with HRP‐conjugated secondary antibodies for 1 h. Western blot images were captured using the Bio‐Rad ChemiDoc system, with signals detected using ECL (Thermo Fisher Scientific). Semi‐quantitative analysis was performed using ImageJ software. The TRNT1 antibody was obtained from Thermo Fisher Scientific, while antibodies for P53, phospho‐P53, caspase‐9, cleaved caspase‐9, and β‐actin were obtained from the Proteintech Group.

### 2.10 Cell Counting Kit‐8 (CCK‐8) Assay

SK‐BR‐3 cells stably overexpressing TRNT1 (OE‐TRNT1) and their corresponding vector control cells were seeded into 96‐well plates at a density of 6000 cells per well. Similarly, MCF‐7 cells transfected with sh‐NC and sh‐TRNT1 were seeded into 96‐well plates at a density of 5000 cells per well. The cells were incubated in a humidified atmosphere with 5% CO_2_ at 37°C. Cell viability was assessed at 24, 48, and 72 h by applying the CCK‐8 reagent (Beyotime) according to the manufacturer's instructions.

### 2.11 Colony Formation Assay

The transfected MCF‐7 cells were seeded in a 6‐well plate at a density of 400 cells per well, and the SK‐BR‐3 cells were seeded at a density of 800 cells per well. The cells were cultured under standard conditions of 37°C in a humidified atmosphere containing 5% CO2 for a duration of 2 weeks. Post‐incubation, cells were fixed with 4% paraformaldehyde (Beyotime, Shanghai, China) for 30 min. Subsequently, cells were stained with 1% crystal violet solution (Beyotime) at room temperature for another 30 min. The stained cells were then washed three times with PBS. Colonies consisting of more than 50 cells were counted for analysis.

### 2.12 Flow Cytometric Analysis of Apoptosis

Transfected cells were harvested using trypsinization without EDTA. The collected cells were washed twice with PBS and subsequently resuspended in 500 μL of Binding Buffer (MultiSciences Biotech Co. Ltd.). Cells were stained at room temperature in the dark for 10 min using the Annexin V‐APC/7‐AAD apoptosis kit (MultiSciences Biotech Co. Ltd.). Flow cytometry was performed using a CytoFLEX flow cytometer (Beckman Coulter Inc.). Data analysis was conducted using Flow Jo software (version 10.8.1).

### 2.13 RNA Sequencing

To explore the mechanisms underlying TRNT1's role in cancer progression, high‐throughput RNA sequencing (RNA‐seq) and gene set enrichment analysis were performed on sh‐NC and sh‐TRNT1‐transfected MCF‐7 cells, as well as vector and OE‐TRNT1‐transfected SK‐BR‐3 cells. RNA‐seq analysis was carried out by Novogene Co. Ltd. To begin, total RNA was extracted from the cells and quality assessed using the Agilent 2100 Bioanalyzer. mRNA was enriched using Oligo (dT) beads, fragmented, and reverse transcribed to generate cDNA. After library preparation (end repair, A‐tailing, adapter ligation, and amplification), libraries were quantified by Qubit 2.0 and Agilent 2100 Bioanalyzer. The effective concentration was confirmed by qRT‐PCR (≥ 1.5 nM). Sequencing was performed on the Illumina platform.

### 2.14 Statistical Analysis

RNA‐seq data were analysed using DESeq2 (version 1.30.1) [19]. Differentially expressed genes were identified based on the filtering criteria of |log2(FoldChange)| ≥ 0.585 and *p*‐value ≤ 0.05. GO functional enrichment and KEGG pathway enrichment analyses were performed using the ‘clusterProfiler’ package. Data visualisation was carried out using the ‘ggplot2’ package. Statistical analysis and visualisation were performed using R software (version 4.2.2) and GraphPad Prism software (version 9.0). Group differences were assessed using the Student's t‐test or one‐way analysis of variance (ANOVA). Pearson and Spearman correlation analyses were employed to evaluate the significance of correlations between two variables. All in vitro experiments were performed independently at least three times. Data are presented as mean ± SD. Statistical significance was considered for *p*‐values < 0.05, with ns indicating no significance, *p < 0.05, **p < 0.01, ***p < 0.001, and ****p < 0.0001.

## 3 Result

### 3.1 Expression Profile of TRNT1

**FIGURE 1:**
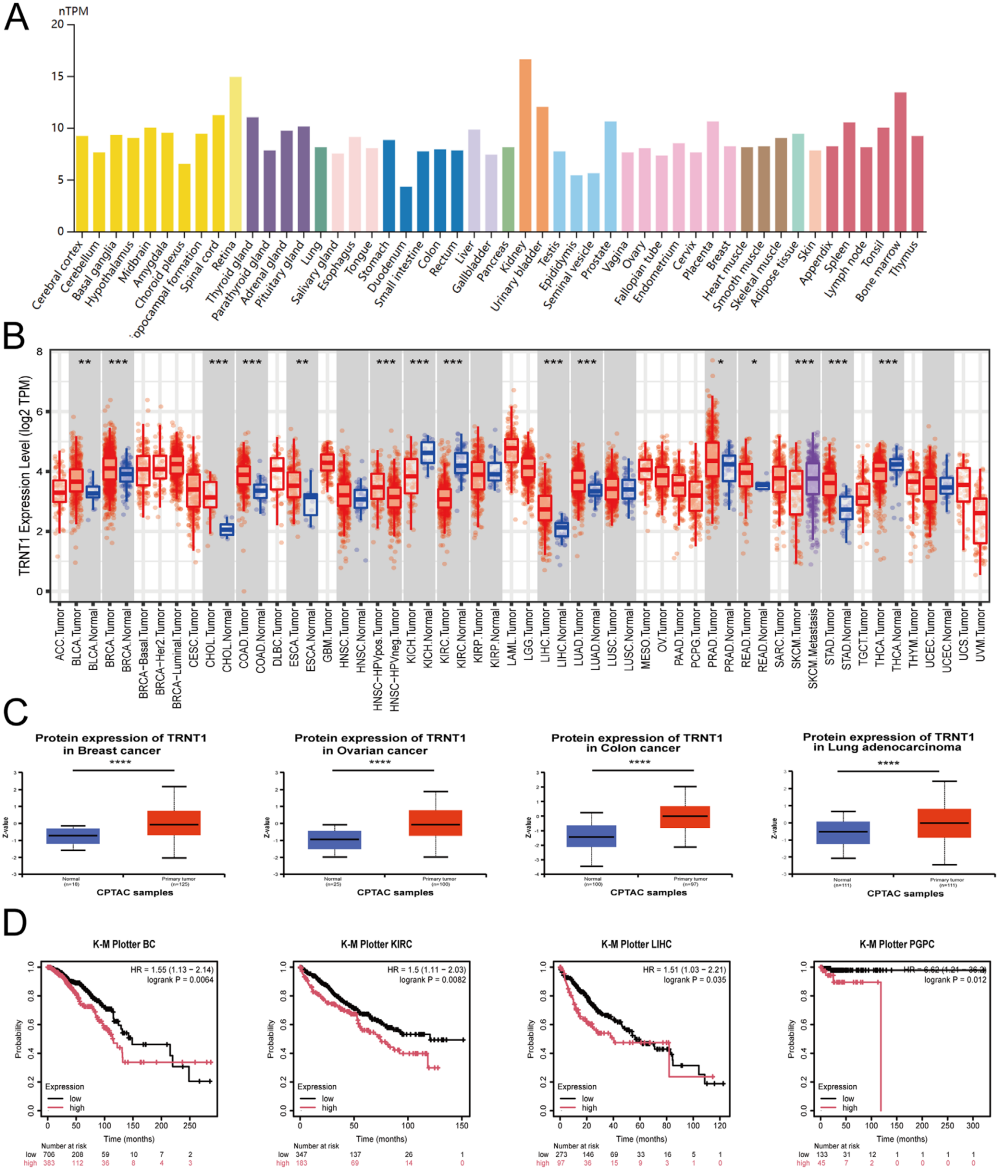
Expression and survival analysis of TRNT1 in various human cancers. (A) TRNT1 mRNA expression profiles in normal human tissues. (B) TRNT1 expression differences between normal and tumour tissues in a pan‐cancer analysis. (C) Differential expression of TRNT1 protein in tumour and normal tissues. (D) Survival prognosis analysis of TRNT1 across multiple cancer types. (*p < 0.05, **p < 0.01, ***p < 0.001, ****p < 0.0001).

To delineate the expression pattern of TRNT1, we used the HPA database to assess TRNT1 mRNA expression levels. The results indicated that TRNT1 expression was highest in the kidney, followed by the retina and bone marrow. Although expression was observed in all tissues, it showed low overall tissue specificity (Figure 1A).

Subsequently, we employed the TIMER2.0 platform to analyse the expression of TRNT1 in various tumour tissues versus normal tissues. As illustrated in Figure 1B, TRNT1 expression levels were significantly elevated in tumour tissues such as bladder urothelial carcinoma (BLCA), BC, cholangiocarcinoma (CHOL), colon adenocarcinoma (COAD), oesophageal carcinoma (ESCA), LIHC, lung adenocarcinoma (LUAD), and prostate adenocarcinoma (PRAD), as well as rectum adenocarcinoma (READ) and stomach adenocarcinoma (STAD) (all *p* < 0.05) compared to their respective control groups. In contrast, TRNT1 expression was significantly downregulated in cancers such as kidney chromophobe (KICH), KIRC, and thyroid carcinoma (THCA) (all *p* < 0.05). Overall, TRNT1 was highly expressed in most tumour tissues.

Further evaluation using the UALCAN database was performed to compare the total protein expression of TRNT1 in tumour versus normal tissues. We found that compared to normal tissues, the total TRNT1 protein expression levels were significantly higher in BC, ovarian cancer (OC), colon cancer (CC), LUAD (Figure 1C), PAAD, and LIHC (all *p* < 0.05) (Figure S1A). Conversely, total TRNT1 protein expression levels were lower in clear cell renal cell carcinoma (RCC) and uterine corpus endometrial carcinoma (UCEC) (Figure S1B).

### 3.2 Survival Analysis Data

Subsequently, we investigated the relationship between TRNT1 gene expression and prognosis across various cancer types. Utilising the Kaplan–Meier Plotter analysis, we identified a significant correlation between TRNT1 expression and patient prognosis in several tumour types. Specifically, elevated TRNT1 expression was associated with poor prognosis in BC, KIRC, LIHC, pheochromocytoma and paraganglioma (PCPG) (Figure 1D). Conversely, high TRNT1 expression was associated with a favourable prognosis in ESCC (all *p* < 0.05; Figure S1C). These findings underscore the potential of TRNT1 as a valuable prognostic biomarker across diverse cancer types. The prognostic significance of TRNT1 expression is contingent upon the cancer type due to tissue‐specific expression patterns.

According to the latest cancer statistics, breast cancer has the highest incidence and mortality rates among all female malignancies, posing a major challenge in the field of global public health [7, 20]. Given the observed significant relationship between TRNT1 expression and breast cancer prognosis, we selected BC as the focus for further investigation.

### 3.3 Genetic Alteration Analysis Data

**FIGURE 2:**
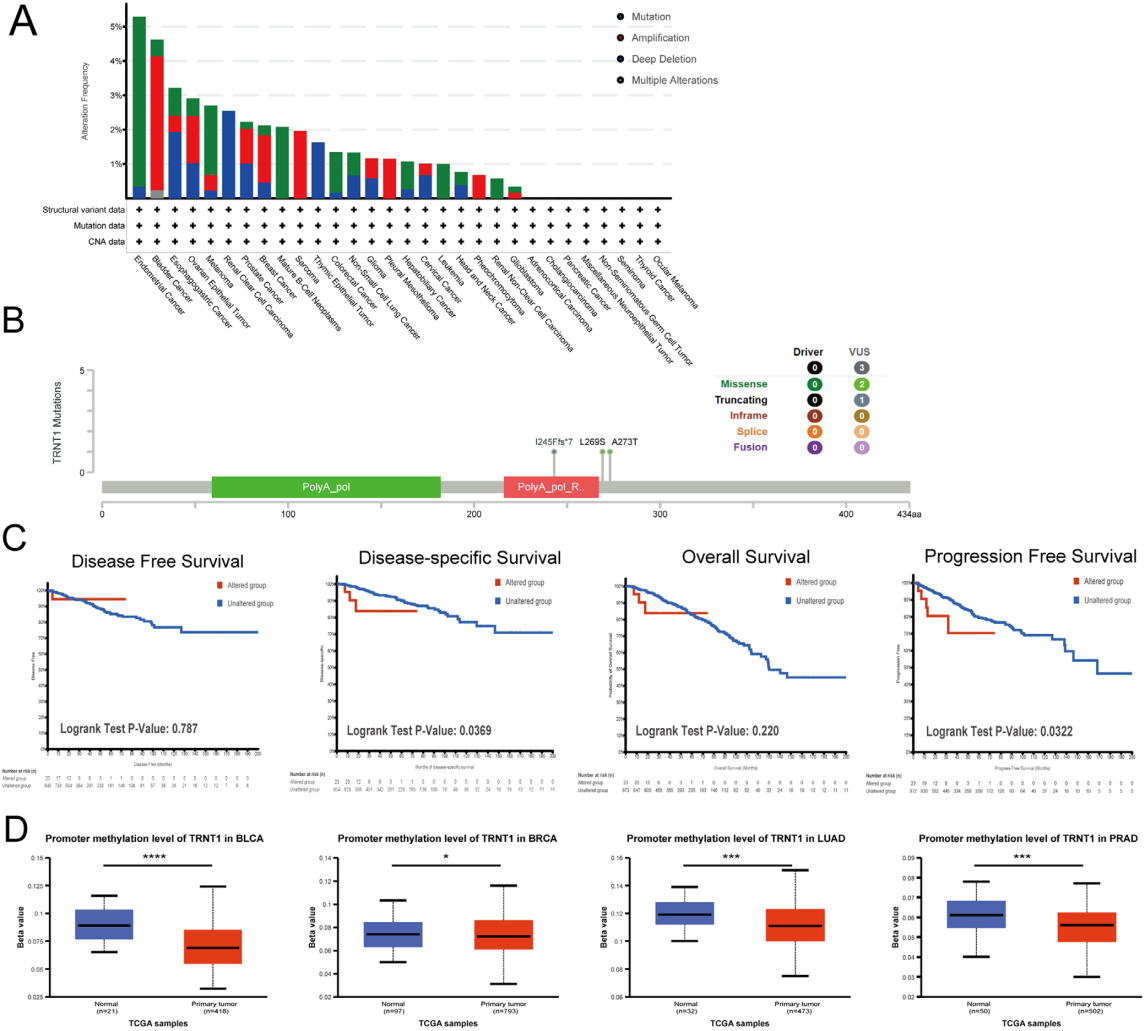
Mutation Status and Promoter Methylation of TRNT1 in Tumours. (A) The landscape of TRNT1 genetic alterations across multiple cancer types. (B) Mutation types and sites of TRNT1 in breast cancer. (C) The correlation between TRNT1 genetic alteration status and DFS (Disease‐Free Survival), DSS (Disease‐Specific Survival), OS (Overall Survival), and PFS (Progression‐Free Survival) in breast cancer. (D) The methylation levels of the TRNT1 promoter in BLCA, BC, LUAD, PRAD. (*p < 0.05, ***p < 0.001, ****p < 0.0001).

Genetic alteration is crucial for the progression of cancer [21]. We analysed the genetic alterations of TRNT1 in tumour samples. Using the cBioPortal database, we investigated the mutation and variant landscape of the TRNT1 gene. Predominantly, ‘Mutation’ type alterations were observed across most cancers. UCEC exhibited the highest TRNT1 variant frequency (> 5%), with ‘Mutation’ as the primary type. In BC, the frequency was 2.12%, with ‘Amplification’ as the main variant (Figure 2A). We further explored the specific mutation types and loci of TRNT1 in breast cancer (Figure 2B), with missense mutations being the primary type of genetic alteration. Additionally, we examined TRNT1 alterations and clinical outcomes in BC. Figure 2C shows that BC patients with TRNT1 alterations had poorer progression‐free (*p* = 0.0322) and disease‐specific survival (*p* = 0.0369) compared to those without, while no significant difference in disease‐free (*p* = 0.787) and overall survival (*p* = 0.220).

### 3.4 Correlation Between TRNT1 DNA Methylation and Expression

DNA methylation is a key epigenetic mechanism regulating gene expression through direct chemical modifications of DNA [22]. We analysed the methylation status of the TRNT1 promoter using UALCAN, and the results revealed that TRNT1 promoter methylation was significantly higher in tumour tissues from CHOL, COAD, head and neck squamous cell carcinoma (HNSC), KIRC, kidney renal papillary cell carcinoma (KIRP), and sarcoma (SARC) (Figure S2A), compared to normal tissues. In contrast, TRNT1 promoter methylation was significantly lower in BLCA, BC, LUAD, PRAD (Figure 2D), READ, and UCEC (Figure S2B). Notably, in BC, TRNT1 showed low methylation and high expression. Low promoter methylation is generally associated with increased gene expression [23], suggesting that TRNT1 may be activated and involved in the development or progression of these cancers. These findings suggest that alterations in TRNT1 promoter methylation and genetic changes may contribute to the dysregulated expression of this gene in various cancers.

### 3.5 Immune Infiltration Analysis

**FIGURE 3:**
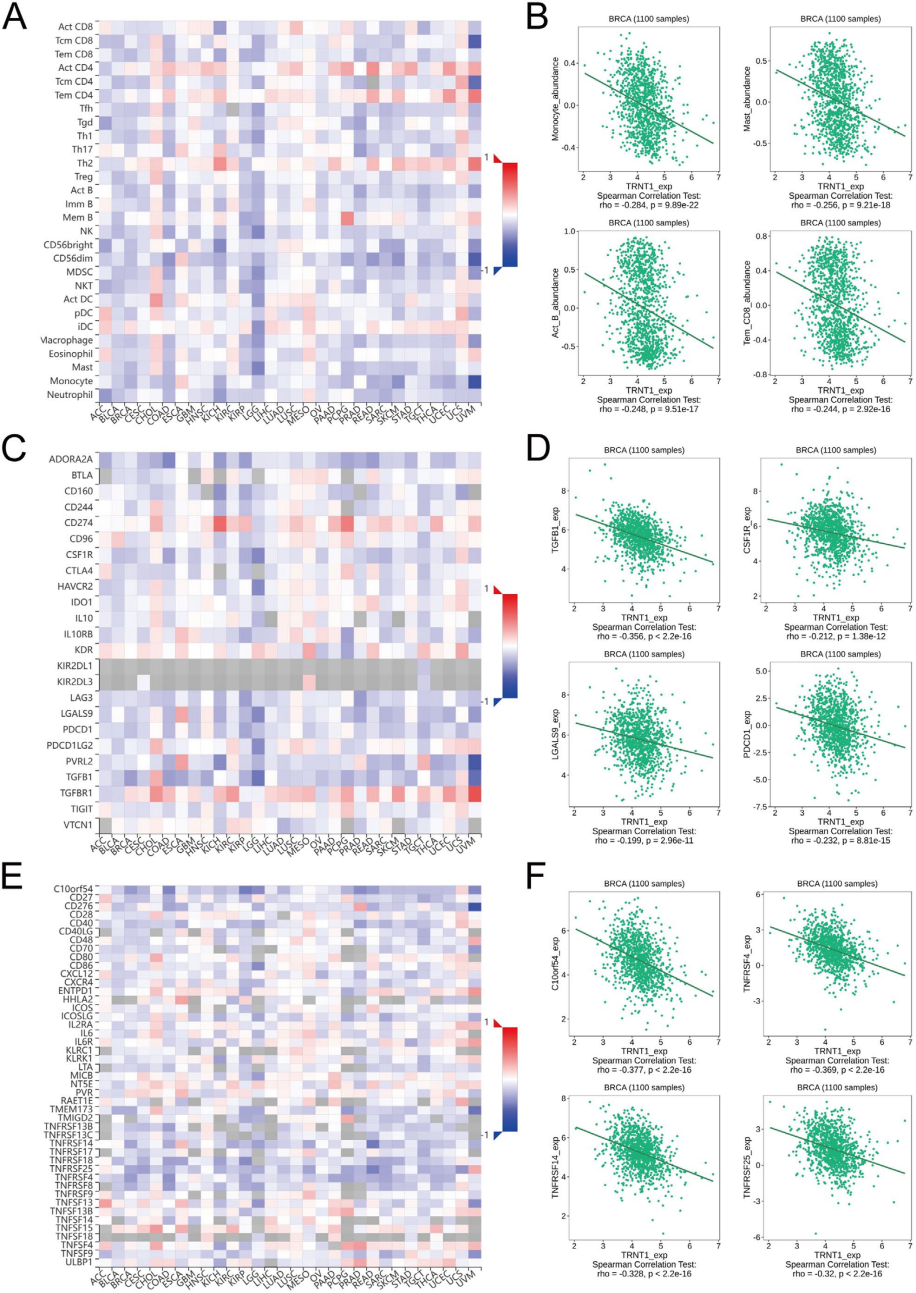
Correlation between TRNT1 expression levels and tumour‐Infiltrating lymphocytes, immunoinhibitors, and immunostimulators in breast cancer. (A) Correlations with tumour‐infiltrating lymphocytes in cancers. (B) Top 4 results of the correlations between TRNT1 expression and lymphocyte infiltration in breast cancer. (C) Correlations with immunoinhibitors in cancers. (D) Top 4 results of the correlations between TRNT1 expression and immunoinhibitors in breast cancer. (E) Correlations with immunostimulators in cancers. (F) Top 4 results of the correlations between TRNT1 expression and immunostimulators in breast cancer.

The immune response plays a crucial role in tumour initiation, progression, and treatment [24]. In‐depth study of the TIME is beneficial for elucidating the mechanisms of immune evasion in cancer and may lead to the identification of potential therapeutic targets for immune intervention. We analysed the correlation between TRNT1 expression and lymphocyte infiltration, immunoinhibitors, and immunostimulators in breast cancer, and for each part, 4 main results were provided. TRNT1 expression was negatively correlated with infiltration of key immune cells (Figure 3A), including monocytes (*r* = −0.284, *p* < 0.001), mast cells (*r* = −0.256, *p* < 0.001), activated B cells (*r* = −0.248, *p* < 0.001), and effector memory CD8 T cells (*r* = −0.244, *p* < 0.001) (Figure 3B). It was also negatively correlated with immunoinhibitors such as TGFB1 (*r* = −0.356, *p* < 0.001) (Figure 3C,D) and immunostimulators like C10orf54 (*r* = −0.377, *p* < 0.001) (Figure 3E,F). In conclusion, patients with high TRNT1 expression may exhibit reduced infiltration of immune effector cells and a potentially lower response to immune checkpoint inhibitor therapy.

### 3.6 TRNT1‐Related Genes Set Enrichment Analysis

**FIGURE 4:**
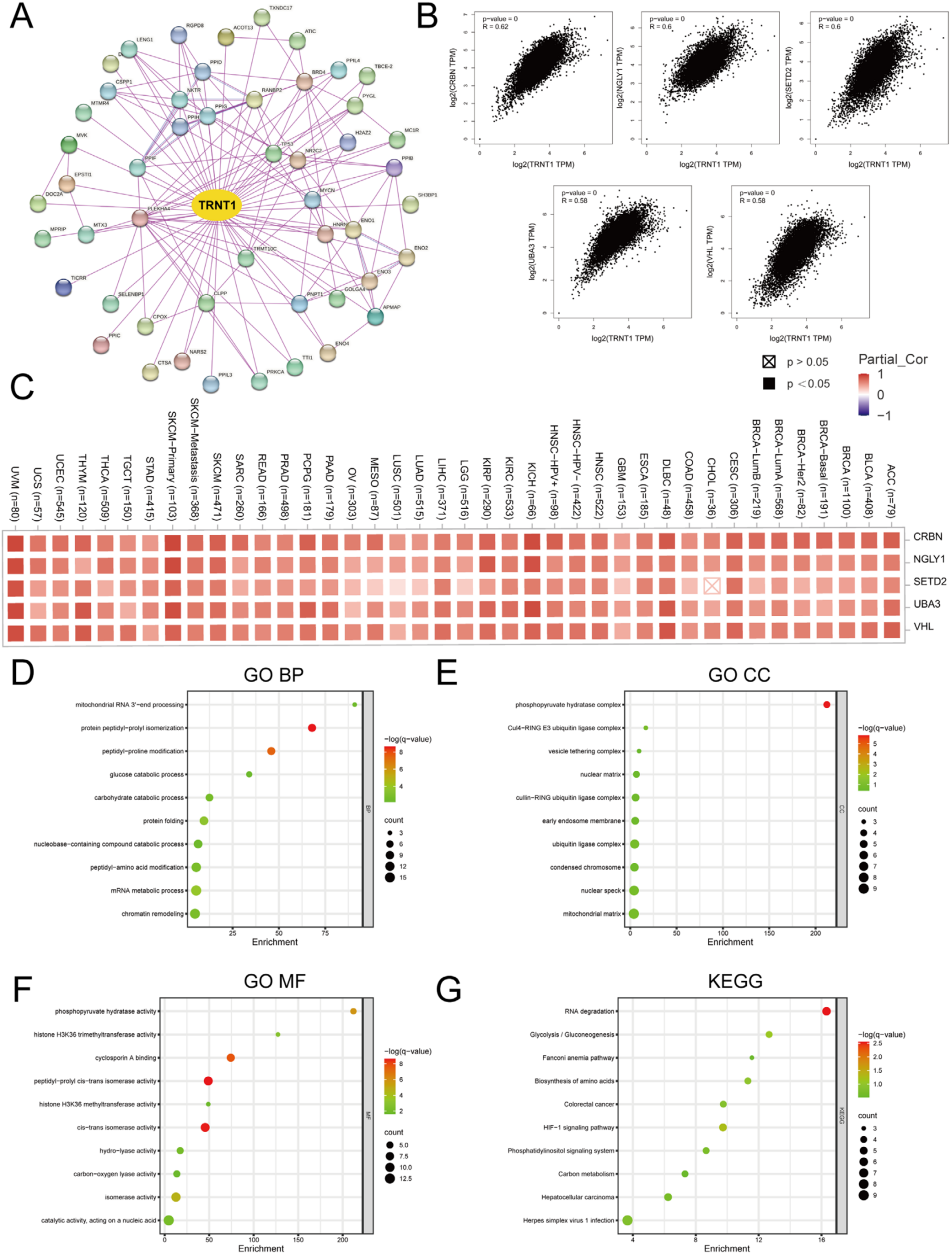
TRNT1‐associated genes and functional enrichment analysis. (A) Protein network map of TRNT1‐interacting proteins with STING tool. (B) Expression correlation between TRNT1 and the top TRNT1‐correlated genes, including CRBN, SETD2, NGLY1, UBA3, and VHL. (C) A heatmap showing correlation coefficients between TRNT1 and CRBN, SETD2, NGLY1, UBA3, and VHL in multiple tumours. (D–G) GO biological process, GO cellular component, GO molecular function and Kyoto Encyclopedia of Genes and Genomes (KEGG) analysis of TRNT1‐related genes.

To investigate the molecular mechanisms of TRNT1 in tumourigenesis, we used the STRING tool to identify 50 TRNT1‐interacting proteins and generated a network diagram illustrating the interactions (Figure 4A). Additionally, we integrated TCGA tumour expression data using the GEPIA2 tool to identify the top 100 genes correlated with TRNT1 expression. Strong positive correlations were observed between TRNT1 and genes such as CRBN, SETD2, NGLY1, UBA3, and VHL (Figure 4B) (*p* < 0.05). Heatmap analysis revealed significant positive correlations between TRNT1 and these five genes across most tumour types (Figure 4C). Subsequently, we performed GO and KEGG enrichment analyses using Metascape, combining data from both datasets. In GO biological process (BP), we found that these genes are likely involved in protein peptidyl‐prolyl isomerisation, peptidyl‐proline modification, and the mRNA metabolic process (Figure 4D). In GO cellular component (CC), these genes were primarily localised to the phosphopyruvate hydratase complex (Figure 4E). In GO molecular function (MF), these genes exhibited activities such as peptidyl‐prolyl cis‐trans isomerase activity, cis‐trans isomerase activity, and cyclosporin A binding (Figure 4F). KEGG analysis indicated that these genes are associated with RNA degradation (Figure 4G).

### 3.7 Validating the Expression and Prognostic Value of TRNT1 in BC

**FIGURE 5:**
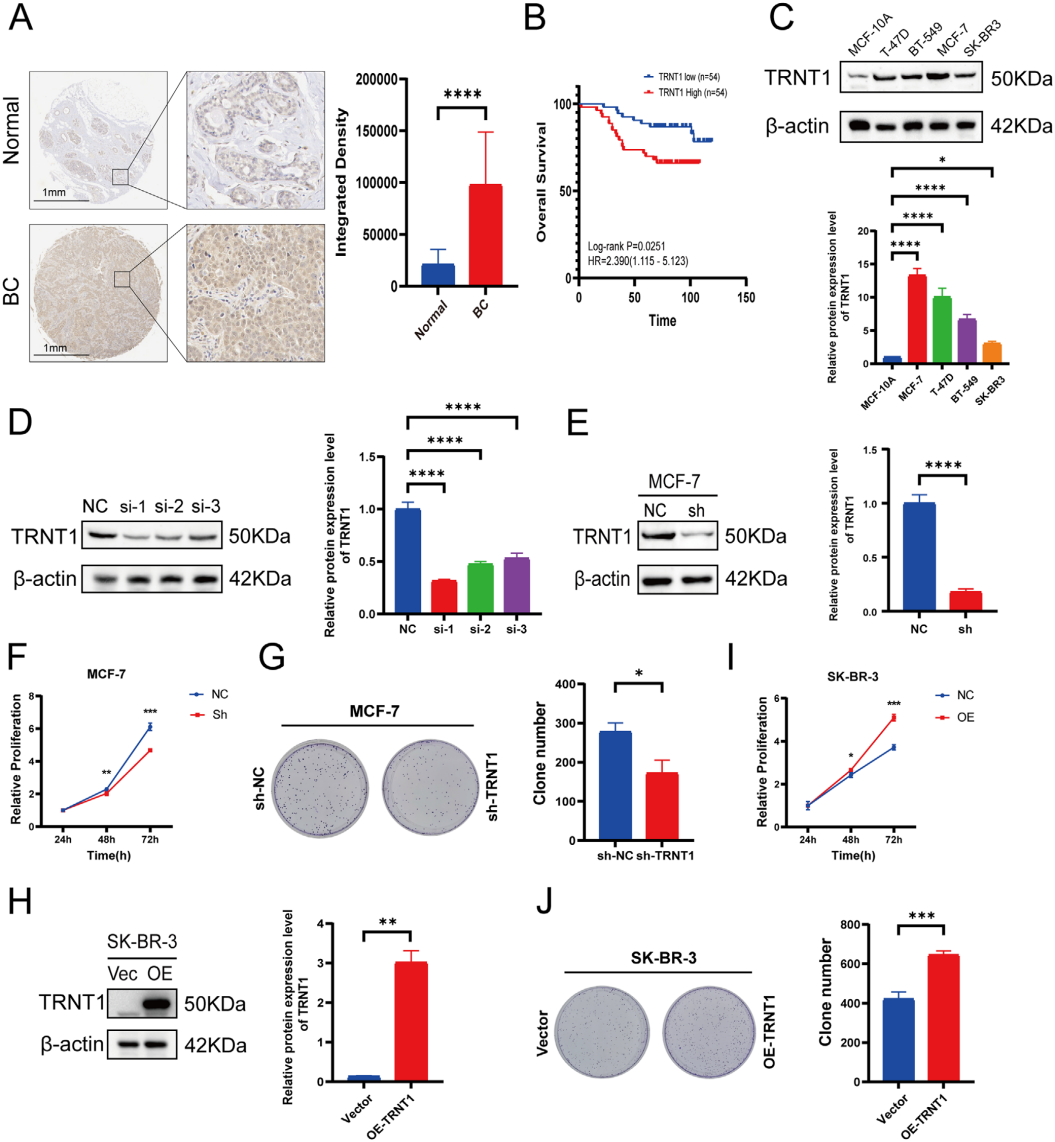
The expression of TRNT1 was increased in BC tissues and affected cell proliferation. (A) IHC staining and analysis for BC sample and adjacent normal tissue. (B) Kaplan‐Meier survival curves analyzing the impact of TRNT1 expression on overall survival (OS) in breast cancer patients. (C) Expression of TRNT1 protein in breast cancer cell lines and normal breast epithelial cells. (D) Western blot analysis of TRNT1 expression in si‐TRNT1 transfected MCF‐7 cells. (E) Western blot analysis of TRNT1 expression in sh‐TRNT1 transfected MCF‐7 cells. (F, G) CCK‐8 and colony formation assay to evaluate cell proliferation in the sh‐TRNT1 group compared to the control group. (H) Western blot analysis of TRNT1 expression in OE‐TRNT1 transfected SK‐BR‐3 cells. (I) CCK‐8 and colony formation assay to evaluate cell proliferation in the OE‐TRNT1 group compared to the control group. The cell experiments were repeated 3 times (*p < 0.05, **p < 0.01, ***p < 0.001, ****p < 0.0001).

To investigate the expression and prognostic significance of TRNT1 in BC, we performed IHC analysis using breast cancer TMAs. This analysis assessed the levels of TRNT1 protein in BC tissues and paired adjacent normal tissues. Statistical analysis revealed that the positive expression rate of TRNT1 protein was significantly higher in BC tissues compared to adjacent non‐cancerous tissues (*p* < 0.001) (Figure 5A). Additionally, no significant differences in TRNT1 expression were observed across different age groups, tumour sizes, AJCC stages, and histologic grades (Table S1). Subsequent Kaplan‐Meier survival analysis of cases with complete follow‐up data stratified patients into high‐ and low‐expression groups based on median TRNT1 expression levels. The high‐expression group exhibited markedly shorter overall survival (OS) than the low‐expression group (log‐rank P = 0.0251) (Figure 5B).

Furthermore, we examined the expression of TRNT1 in BC cell lines using Western blot. We included the normal breast epithelial cell line MCF‐10A, as well as various BC cell lines, including BT‐549, MCF‐7, T‐47D, and SK‐BR‐3. Compared to MCF‐10A cells, TRNT1 protein expression was significantly upregulated in all BC cell lines. Notably, TRNT1 expression was highest in MCF‐7 cells and lowest in SK‐BR‐3 cells (Figure 5C).

### 3.8 The Biological Functions of TRNT1 in Breast Cancer Cells

We investigated the effects of TRNT1 on BC cells in vitro. Based on the expression levels of TRNT1 in various BC cell lines, we selected MCF‐7 cells, which exhibited the highest TRNT1 expression, for siRNA transfection. Western blot analysis revealed that all three TRNT1 siRNAs significantly downregulated TRNT1 protein expression in MCF‐7 cells, with siRNA‐1 showing the highest knockdown efficiency (Figure 5D). Using the siRNA‐1 sequence with the best knockdown efficiency, we constructed a corresponding shRNA lentivirus. After infecting MCF‐7 cells with the lentivirus, we again analysed TRNT1 expression by Western blot. The results confirmed a significant reduction in TRNT1 protein levels in MCF‐7 cells following shRNA‐mediated transfection (Figure 5E), indicating the successful construction of TRNT1 knockdown MCF‐7 cells. To assess the impact of TRNT1 knockdown on cell proliferation, we performed CCK‐8 and colony formation assays. CCK‐8 results (Figure 5F) showed that, compared to MCF‐7 (shRNA‐NC) cells, MCF‐7 (shRNA‐TRNT1) cells exhibited significantly lower cell viability at 48 and 72 h, with statistical significance. Colony formation assays further revealed that the number of colonies formed by MCF‐7 (shRNA‐TRNT1) cells was significantly fewer than that of MCF‐7 (shRNA‐NC) cells (Figure 5G). These findings suggest that TRNT1 knockdown inhibits the proliferative capacity of BC cells.

We also constructed TRNT1 overexpressing SK‐BR‐3 cells via lentiviral transfection. Successful overexpression of TRNT1 in SK‐BR‐3 cells was confirmed by Western blot analysis (Figure 5H). To evaluate the effect of TRNT1 overexpression on cell proliferation, we conducted CCK‐8 and colony formation assays. CCK‐8 results (Figure 5I) demonstrated that, compared to the control group (Vector), SK‐BR‐3 cells with TRNT1 overexpression (OE‐TRNT1) exhibited significantly higher cell viability at 48 and 72 h, with statistical significance. Colony formation assays showed that the OE‐TRNT1 group formed significantly more colonies than the Vector group (Figure 5J). These results indicate that TRNT1 overexpression promotes the proliferative capacity of BC cells.

### 3.9 Functional Enrichment Analysis of TRNT1 in BC

**FIGURE 6:**
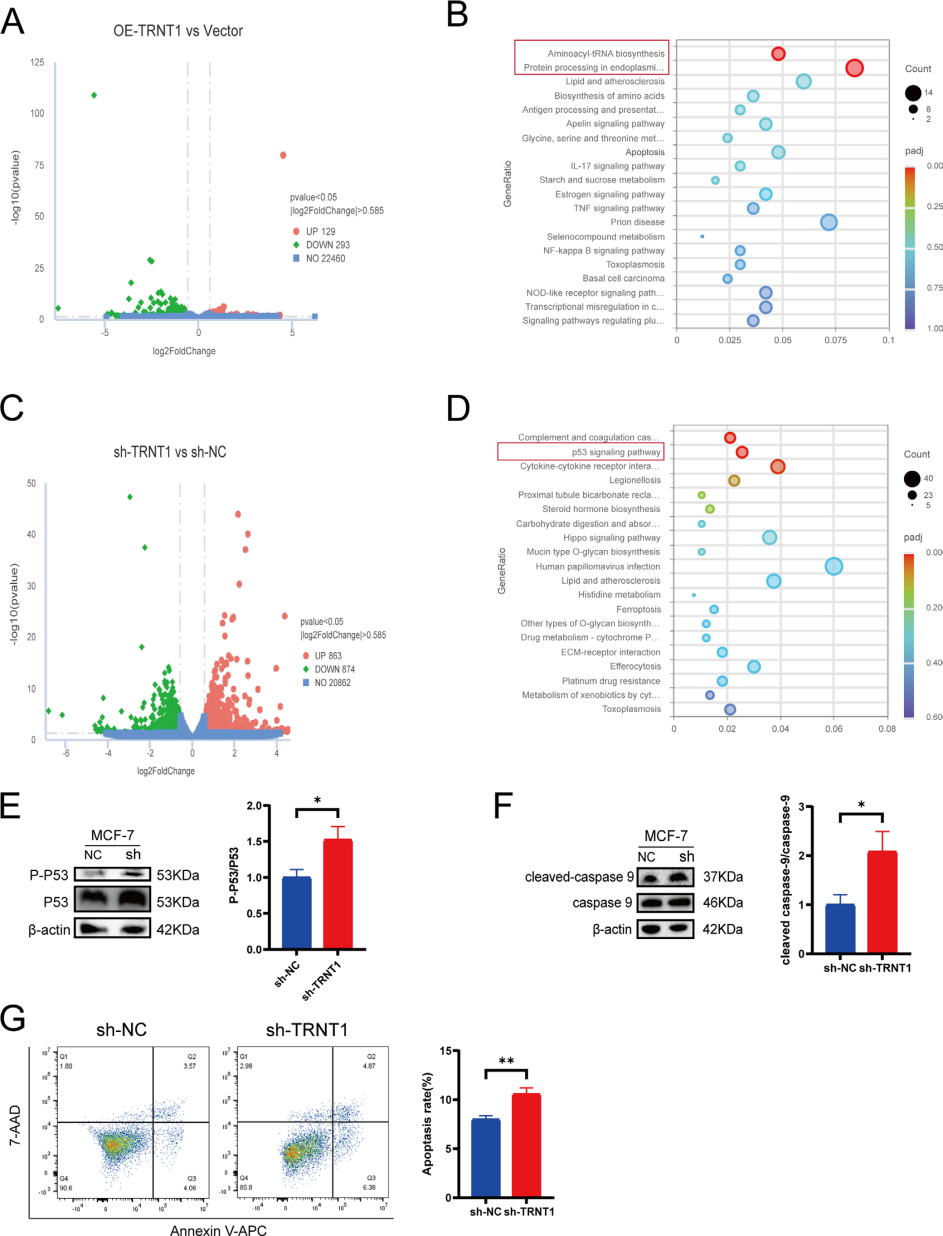
Validating the potential signalling pathways of TRNT1. (A) Volcano plot of differentially expressed genes in SK‐BR‐3 cells after overexpression of TRNT1. (B) KEGG analysis of differentially expressed genes between vector and OE‐TRNT1. (C) Volcano plot of gene expression differences in MCF‐7 cells after knockdown of TRNT1. (D) KEGG analysis of differentially expressed genes between sh‐NC and sh‐TRNT1. (E‐F) WB results demonstrated changes in P53 signalling pathways following knockdown of TRNT1. (G) Flow cytometry analysis of apoptosis changes in MCF‐7 cells after TRNT1 knockdown. The cell experimenta were repeated 3 times (*p < 0.05, **p < 0.01).

To elucidate the potential mechanisms by which TRNT1 exerts its effects in BC cells, we conducted RNA‐seq analysis on SK‐BR‐3 cells overexpressing TRNT1 (OE‐TRNT1) versus vector control and on MCF‐7 cells with TRNT1 knockdown (sh‐TRNT1) versus non‐targeting control (sh‐NC). The results revealed that, compared to the Vector group, the OE‐TRNT1 group exhibited 422 differentially expressed genes (DEGs). Specifically, 129 genes were upregulated, and 293 genes were downregulated in the context of TRNT1 overexpression (Figure 6A). Subsequently, we performed gene set enrichment analysis to identify enriched KEGG signalling pathways. The analysis indicated that the DEGs were predominantly enriched in the aminoacyl‐tRNA biosynthesis pathway (*p* < 0.05) and the protein processing in endoplasmic reticulum pathway (*p* < 0.05) (Figure 6B). These findings suggest that TRNT1 overexpression may enhance cellular protein synthesis, thereby promoting cell proliferation.

In contrast, when comparing the sh‐TRNT1 group to the sh‐NC group, a total of 1737 DEGs were identified, with 863 genes being upregulated and 874 genes downregulated (Figure 6C). KEGG enrichment analysis demonstrated that these DEGs were significantly enriched in the p53 signalling pathway (*p* < 0.01) (Figure 6D). These results imply that the downregulation of TRNT1 may inhibit tumour progression by activating the p53 signalling pathway.

### 3.10 Validating the Impact of TRNT1 on the p53 Signalling Pathway

Given the critical role of the p53 signalling pathway in the regulation of tumour progression [25], we hypothesised that TRNT1 might modulate BC progression through this pathway. To further validate the impact of TRNT1 knockdown on BC cells via the p53 pathway, we performed WB on cells from both the shRNA‐TRNT1 and sh‐NC groups to examine protein expression patterns. Our findings revealed that TRNT1 knockdown significantly increased the ratio of phosphorylated p53 (p‐p53) to total p53 (Figure 6E). Additionally, there was an upregulation in the expression of cleaved caspase‐9 relative to total caspase‐9 (Figure 6F).

These results indicate that TRNT1 knockdown activates the phosphorylation of p53, and the presence of cleaved caspase‐9 further substantiates the initiation of downstream apoptotic signalling, with cleaved caspase‐9 serving as a clear molecular hallmark of apoptosis. Subsequently, to investigate the effect of TRNT1 silencing on cell apoptosis, we conducted additional functional assays. Flow cytometry analysis for apoptosis demonstrated that the proportion of apoptotic cells was significantly increased in the shTRNT1 group (Figure 6G).

These findings suggest that TRNT1 knockdown activates the p53 signalling pathway and promotes apoptosis.

## 4 Discussion

TRNT1, an essential enzyme catalysing the addition of the terminal CCA trinucleotide to all mature tRNAs, plays a critical role in RNA metabolism [26]. While CCA addition has been well studied in prokaryotes and lower eukaryotes, the association between TRNT1 and human diseases has only recently been recognised [3, 5, 27]. BC has surpassed lung cancer to become the most commonly diagnosed cancer, posing a significant public health challenge [28], highlighting the need for further research into effective biomarkers and therapeutic targets. A comprehensive literature search revealed no prior studies that have analysed TRNT1 from a pan‐cancer perspective, and the biological impact of TRNT1 on cancer cells remains unclear. Therefore, using data from TCGA and CPTAC databases, we conducted an integrated exploration of TRNT1's potential role in tumourigenesis, focusing on gene expression, genetic alterations, DNA methylation, and immune infiltration. Our investigation primarily concentrated on TRNT1 expression and function in BC. Our findings suggest that TRNT1 may play a pivotal role in the development of BC and has the potential to serve as a novel prognostic biomarker for BC.

The results of this study indicate that TRNT1 is overexpressed at both the mRNA and protein levels in a variety of tumours, including BC, a finding that was further validated in BC tissues and cell lines. However, survival analysis revealed that the prognostic value of TRNT1 expression is cancer‐type dependent. Previous studies have shown that TRNT1 expression is downregulated in African American patients compared to European populations, and that African American individuals have higher incidence and mortality rates for CRC [6]. This suggests that TRNT1 may play a suppressive role in tumour progression in certain cancers. In contrast, our study found that high TRNT1 expression in BC is associated with poor prognosis, suggesting that the biological function of TRNT1 is not universal but may vary across different cancer types.

Genetic alteration analysis revealed significant heterogeneity in TRNT1 genetic alterations across different cancer types, highlighting the diverse nature of TRNT1 mutations in various tumour contexts. The identification of specific missense mutations, such as A273T, particularly in BC, suggests that mutations at particular loci may play a critical role in the loss or gain of TRNT1 function. Moreover, BC patients harbouring TRNT1 alterations exhibit poorer prognosis in terms of disease progression and disease‐specific survival, underscoring the potential of TRNT1 alterations as prognostic biomarkers. However, no significant differences were observed in terms of disease‐free survival and overall survival, which may indicate that the impact of TRNT1 mutations is more pronounced in the early stages or specific phases of the disease. Epigenetic modifications, particularly DNA methylation, play a crucial role in tumourigenesis and gene expression regulation [29]. Hypomethylation of oncogene promoters is considered a driving factor in tumour initiation and progression, potentially leading to gene overexpression and activation of downstream signalling pathways [30, 31]. Our analysis identified significant hypomethylation of the TRNT1 promoter in tumour tissues compared to adjacent normal tissues across multiple cancer types. In BC, TRNT1 promoter methylation was notably lower than in adjacent normal tissue, while TRNT1 expression was elevated in BC tissues compared to normal tissues. These findings suggest that promoter hypomethylation may be a key mechanism underlying TRNT1 overexpression in BC.

TIME plays a critical role in tumour initiation and progression [32]. In this study, we examined the relationship between TRNT1 and TIME and found a negative correlation between TRNT1 expression and immune cells, such as monocytes, mast cells, effector memory CD8+ T cells, and activated B cells In early breast cancer, M1‐type Tumour‐Associated Macrophages, derived from monocytes, activate anti‐tumour immunity and promote tumour cell clearance through the release of factors like IL‐6, IL‐12, and TNF‐α; reduced monocyte infiltration weakens this anti‐tumour immune surveillance [33]. Mast cell‐derived TNF‐α enhances the function of CD8+ dendritic cells and promotes T cell activation. Additionally, mast cells directly kill tumour cells through the release of TNF‐α and reactive oxygen species [34]. The infiltration level of effector memory CD8+ T cells in breast cancer is positively correlated with patient prognosis, while insufficient infiltration often indicates a poor response to immune therapy [35]. Activated B cells cooperate with T cells to target tumours through antigen presentation. A reduction in activated B cells may lead to impaired formation of tertiary lymphoid structures (TLS), which predicts poor prognosis [36]. Immune cell infiltration is crucial for tumour recognition and elimination [24], suggesting that TRNT1 may support tumour immune evasion by reducing immune cell infiltration. High TRNT1 expression was significantly negatively correlated with immunosuppressive factors, as well as with immune stimulators. This dual negative correlation, combined with the characteristic reduction in immune cell infiltration, collectively suggests that in breast cancer patients with high TRNT1 expression, their tumour microenvironment may present a state of insufficient immune effector cell recruitment and immune regulatory factor imbalance. This leads to a weakened anti‐tumour immune response, resulting in a ‘cold’ tumour, which may subsequently reduce the tumour's response potential to immune therapies [37].

Furthermore, using the GEPIA2 tool, we identified genes closely associated with TRNT1. The significant positive correlations between TRNT1 and these genes suggest they may function synergistically within shared biological pathways. GO and KEGG enrichment analyses of TRNT1‐associated genes revealed their potential involvement in critical processes such as protein post‐translational modifications and RNA processing. These findings imply that TRNT1 may influence tumourigenesis by modulating fundamental aspects of cellular protein synthesis and metabolic homeostasis.

To reliably assess the role of TRNT1 in BC, we conducted a series of experiments. In vitro, we confirmed that TRNT1 expression was significantly elevated in multiple BC cell lines compared to normal breast epithelial cells. Moreover, overexpression of TRNT1 promoted cell proliferation, while TRNT1 knockdown inhibited cell growth, supporting the notion that TRNT1 plays a pro‐tumorigenic role in cancer cell development. Further transcriptomic analysis and KEGG pathway enrichment analysis revealed that TRNT1 overexpression significantly enriched pathways related to aminoacyl‐tRNA biosynthesis and protein processing in the endoplasmic reticulum. A metabolomics study has shown that the aminoacyl‐tRNA biosynthesis pathway is upregulated in gastric cancer, contributing to metabolic dysregulation and correlating with poor prognosis and tumour metastasis [38]. These findings suggest that TRNT1 may support tumour growth by modulating tumour metabolism and promoting protein synthesis pathways. TRNT1 knockdown was associated with significant enrichment of the p53 signalling pathway, suggesting that downregulation of TRNT1 may enhance p53 activity, thereby inhibiting tumour progression. P53 is a critical mediator of various signalling pathways that influence cancer progression [39], playing a pivotal role in regulating both cell proliferation and apoptosis [40]. Cell death induced through the p53 pathway is executed by caspase proteases [41]. Caspases, particularly caspase‐9, play a key role in apoptosis, with caspase‐9 acting as an initiator that triggers downstream caspase‐mediated cell death [42]. Our Western blot and flow cytometry‐based apoptosis assays further demonstrated that TRNT1 knockdown activates the p53 signalling pathway and promotes apoptosis in BC cells. These results highlight the importance of TRNT1 in regulating critical signalling pathways that contribute to tumour growth and cell survival. Enrichment analyses of TRNT1‐associated genes and breast cancer RNA‐seq both indicate its strong involvement in protein modification and RNA regulation. TRNT1 appears to modulate protein activity through post‐translational mechanisms, as supported by both pan‐cancer and breast cancer‐specific analyses [43]. In RNA‐related processes, it influences stability and translation accuracy via pathways such as RNA degradation and aminoacyl‐tRNA biosynthesis [43, 44]. These mechanisms collectively regulate gene expression and protein synthesis.

It is important to acknowledge the limitations of our study. First, although preliminary evidence suggests TRNT1 regulates breast cancer progression through the p53 pathway, systematic characterisation of its downstream effector molecules is lacking, requiring further rigorous validation. Secondly, this study mainly relies on bioinformatics analysis data and in vitro cell experiments. While these data provide initial evidence for the potential role of TRNT1 in breast cancer, the lack of in vivo animal model validation remains a limitation. Furthermore, our research only provides an initial investigation into the expression and function of TRNT1 in breast cancer, without further exploration of its role in different breast cancer subtypes. Therefore, future studies should focus on investigating the role of TRNT1 in various breast cancer subtypes and conducting animal model experiments to more accurately assess its role in the onset and progression of breast cancer.

## Author Contributions

**Xinwei Li:** conceptualization (equal), data curation (equal), validation (equal), visualization (equal), writing – original draft (equal). **Yue Meng:** conceptualization (equal), project administration (equal), writing – review and editing (equal). **Bing Gu:** conceptualization (equal), funding acquisition (equal).

## Ethics Statement

Ethical approval for the study of the tissue microarray slide was granted by the Clinical Research Ethics Committee, Outdo Biotech (Shanghai, China) (Ethical Approval Number: SHYJS‐CP‐1410018, SHYJS‐CP‐1901010).

## Consent

All authors have approved the manuscript for submission.

## Conflicts of Interest

The authors declare no conflicts of interest.

## Supporting information


**Figure S1:** Protein expression levels and survival analysis of TRNT1. (A, B) TRNT1 protein expression levels in PAAD, LIHC, RCC and UCEC. (C) Survival analysis of TRNT1 in ESCC. (*p < 0.05, **p < 0.01, ***p < 0.001, ****p < 0.0001).

**Figure S2:** Methylation Status of the TRNT1 Promoter in Tumours. (A, B) The methylation levels of the TRNT1 promoter in CHOL, COAD, HNSC, KIRC, KIRP, SARC, READ, and UCEC. (*p < 0.05, **p < 0.01, ***p < 0.001, ****p < 0.0001).

**Table S1:** Association between TRNT1 expression and clinicopathological characteristics of breast cancer patients.

## Data Availability

The data that support the findings of this study are available from the corresponding author upon reasonable request.
