# Supplementary figures and images for: Comprehensive Pan‐Cancer Analysis of TRNT1 as a Potential Biomarker for Breast Cancer
Source: J Cell Mol Med. 2025 Sep 26;29(18):e70853. doi: 10.1111/jcmm.70853 (PMC12464730; doi:10.1111/jcmm.70853)

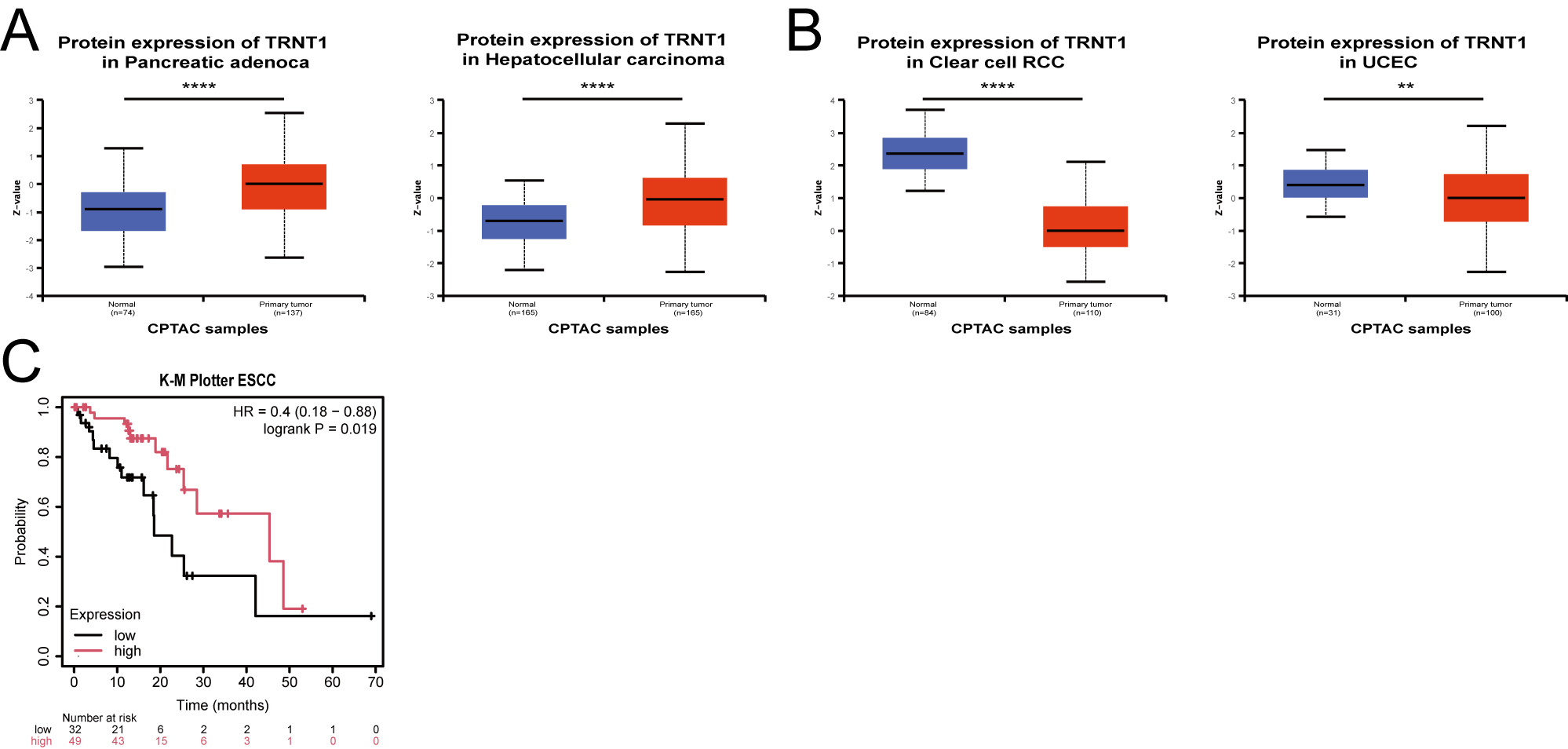

Supplement: Supplementary file 1 — Figure S1: Protein expression levels and survival analysis of TRNT1. (A, B) TRNT1 protein expression levels in PAAD, LIHC, RCC and UCEC. (C) Survival analysis of TRNT1 in ESCC. (*p < 0.05, **p < 0.01, ***p < 0.001, ****p < 0.0001). [file JCMM-29-e70853-s001.tif]

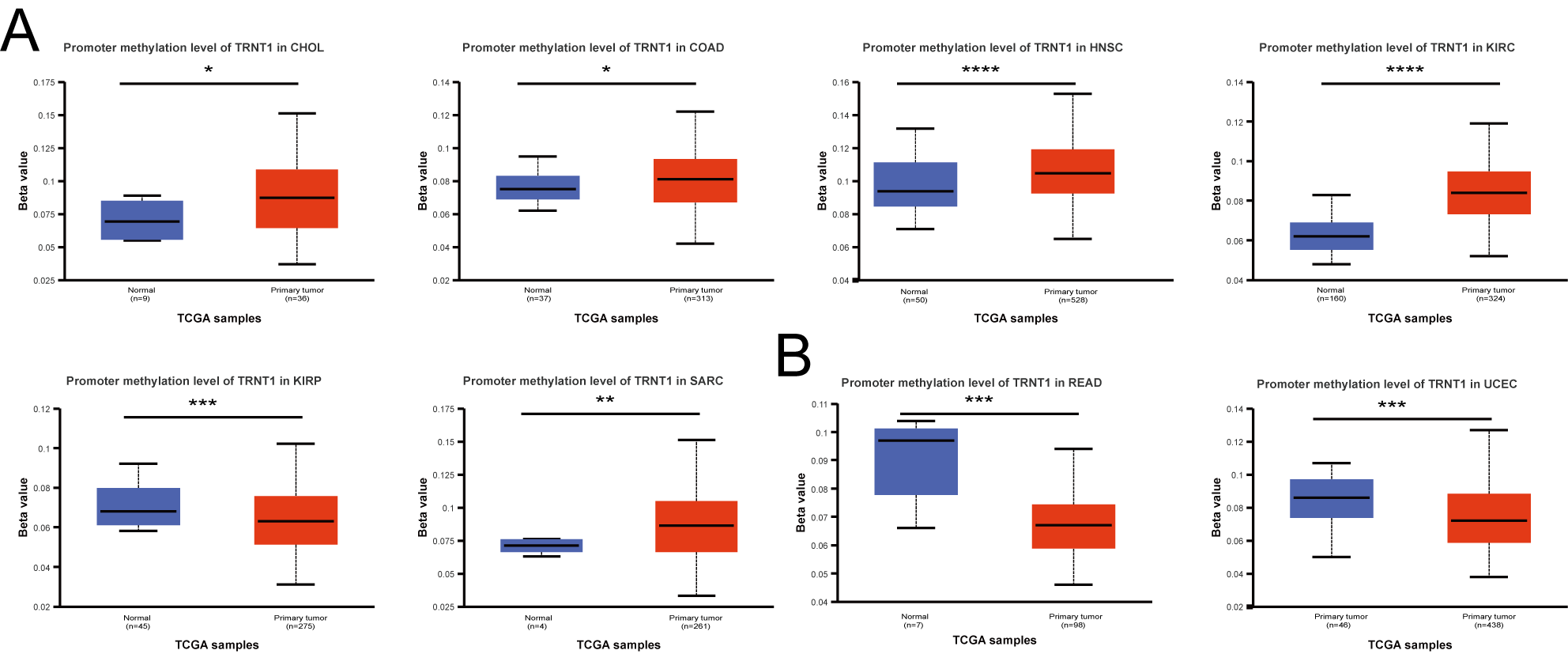

Supplement: Supplementary file 2 — Figure S2: Methylation Status of the TRNT1 Promoter in Tumours. (A, B) The methylation levels of the TRNT1 promoter in CHOL, COAD, HNSC, KIRC, KIRP, SARC, READ, and UCEC. (*p < 0.05, **p < 0.01, ***p < 0.001, ****p < 0.0001). [file JCMM-29-e70853-s003.tif]
