# Supplementary material for: Comprehensive Pan‐Cancer Analysis of TRNT1 as a Potential Biomarker for Breast Cancer
Source: J Cell Mol Med. 2025 Sep 26;29(18):e70853. doi: 10.1111/jcmm.70853 (PMC12464730; doi:10.1111/jcmm.70853)
Supplement: Supplementary file 3 — Table S1: Association between TRNT1 expression and clinicopathological characteristics of breast cancer patients. [file JCMM-29-e70853-s002.docx]

Table S1. Association between TRNT1 expression and clinicopathological characteristics of breast cancer patients

| Parameter | N | H-score | *p*-Value |
| --- | --- | --- | --- |
| Age |  |  |  |
| <50 | 41 | 39.65±17.10 | 0.8303 |
| ≥50 | 98 | 39.00±15.82 |  |
| AJCC stage |  |  |  |
| 0-2 | 97 | 40.53±16.41 | 0.1157 |
| 3-4 | 33 | 35.34±15.76 |  |
| Histologic grade |  |  |  |
| 1-2 | 78 | 38.72±17.05 | 0.6152 |
| 3 | 60 | 40.12±14.94 |  |
| Lymph node metastasis |  |  |  |
| N0 | 63 | 42.91±16.55 | 0.0191 |
| N1-3 | 74 | 36.52±15.00 |  |
| Tumor size |  |  |  |
| <2 | 17 | 36.65±14.55 | 0.4894 |
| ≥2 | 122 | 39.55±16.38 |  |
| T stage |  |  |  |
| T0-2 | 114 | 39.60+16.93 | 0.9606 |
| T3-4 | 16 | 39.39±12.49 |  |

Data are mean ± SD. AJCC, American Joint Committee on Cancer.
